# The Emerging Role of Gut Microbiota in Inflammatory Skin Diseases: A Systematic Review

**DOI:** 10.1111/exd.70234

**Published:** 2026-03-08

**Authors:** Andrea Malgesini, Matteo Domenico Marsiglia, Elisa Borghi, Angelo V. Marzano, Gianluca Nazzaro

**Affiliations:** ^1^ Department of Pathophysiology and Transplantation University of Milan Milan Italy; ^2^ Dermatology Unit Fondazione IRCCS Ca' Granda Ospedale Maggiore Policlinico Milan Italy; ^3^ Department of Health Sciences University of Milan Milan Italy

**Keywords:** atopic dermatitis, gut microbiome, hidradenitis suppurativa, psoriasis

## Abstract

The human gut microbiota is involved in immune regulation, metabolism, and skin homeostasis. In recent years, gut microbiota alterations have been linked with several inflammatory skin disorders, such as atopic dermatitis (AD), psoriasis, and hidradenitis suppurativa (HS). This systematic review synthesises current evidence on gut microbiota composition and functional alterations in these dermatoses. A comprehensive literature search was conducted in the PubMed database, identifying studies from inception to January 2025. Eligible studies included human observational, interventional, and genetic studies investigating gut microbiota alterations in AD, psoriasis, or HS, using microbiome profiling or genetic causal‐inference approaches. Studies lacking control groups or relying on culture‐based techniques were excluded. Sixty‐two studies were included: 38 on AD, 22 on psoriasis and 5 on HS, with three addressing more than one disease. In AD, most studies focused on paediatric populations, leaving a knowledge gap regarding adult‐specific data. Reduced alpha‐diversity and decreased abundance of *Faecalibacterium prausnitzii* , *Bifidobacterium* spp., and *Akkermansia muciniphila* were recurrent findings. In psoriasis, in addition to dysbiosis, microbial metabolic pathways were also found to be altered. In HS, data remain limited, but increased *Ruminococcus gnavus* and reduced alpha‐diversity have been reported, mirroring findings in inflammatory bowel diseases. Gut microbiota has been increasingly implicated in skin inflammation. Despite advances in microbiota analysis, significant gaps remain—especially in adult AD and HS. Future research should prioritize standardised methodologies, larger and more diverse cohorts, and leverage emerging tools such as Mendelian randomization and AI‐based models to develop precision medicine interventions.

## 1 Introduction

The human gut harbours a vast and dynamic community of microorganisms including bacteria, viruses and eukaryotic species that collectively form the gut microbiota [1]. This ecosystem coevolves with the host and contributes to general health in a multitude of ways, including digestion, immunity and creation of vital compounds that the body cannot synthesise on its own [2].

Microbial colonisation begins at birth and stabilises during early childhood, shaping the host's immune and metabolic profile [3]. The composition of the gut microbiota remains relatively stable in adulthood but can be profoundly influenced by factors such as drugs, infections, and disease states [4].

Diet also plays a critical role in shaping the composition, function, and diversity of the gut microbiota. Notably, a one‐year Mediterranean dietary intervention in elderly individuals can positively affect the gut microbiota, leading to improved markers of lower frailty, cognitive function, and reduced inflammation [5].

When the adult‐like state of gut microbiota is reached, the taxonomic composition of the human microbiota varies tremendously across individuals. However, its functional capacity is highly conserved—implying an ecological property known as functional redundancy.

The gut microbiome, defined as the collective genome of microorganisms inhabiting the gastrointestinal tract, plays a fundamental role in human metabolism by producing enzymes not encoded by the human genome. These enzymes enable the breakdown of complex polysaccharides and polyphenols, as well as the synthesis of vitamins. Short chain fatty acids (SCFAs) not only are a source of energy for human colonocytes but also play a wide range of metabolic functions. The three most abundant SCFAs are acetate, propionate, and butyrate and are products of the bacterial fermentation of undigested carbohydrates in the intestine. In particular, butyrate enhances the intestinal barrier by regulating the assembly of tight junctions [5] and exerts potent immunomodulatory effects by inducing regulatory T cells (Treg) differentiation and controlling inflammation [6]. SCFAs can directly activate G‐coupled receptors and inhibit histone deacetylases, thus affecting various physiological processes and contributing to health and disease [7]. Moreover, they can be transported from the intestine to the skin via peripheral circulation, regulating both innate and adaptive immune cells and promoting keratinocyte metabolism and differentiation [8].

There are bidirectional influences between gut microbiota and the immune system. The loss of beneficial functions of commensal microorganisms can result in activation of the immune system, with a causal or contributory role in many diseases. It is still unknown whether changes in gut microbiota are primary events or secondary consequences of the dermatoses themselves [9].

In recent years, new data have accumulated on the role of gut microbiota in systemic diseases. In particular, the existence of a gut‐skin axis has been supported by experimental and clinical studies: an unbalanced gut microbial community can negatively impact the homeostasis of the skin. For instance, some of the skin manifestations associated with Inflammatory Bowel Diseases (IBD) may correlate with the amount of gut inflammation [10]. The link between these two apparently distant sites has not yet been fully clarified, but the immune and the endocrine systems may play a relevant role. Intestinal dysbiosis represents the primary trigger of gut‐skin axis imbalance: the overgrowth of specific bacterial species can lead to T‐cell activation while downregulating immunosuppressive cytokines and Treg, thereby reducing immune tolerance to commensal microbes [11]. Chronic inflammation in either the gut or the skin can ultimately become self‐perpetuating and contribute to disease maintenance.

Evidence from inflammatory skin conditions further highlights the importance of this axis. For example, chronic spontaneous urticaria has been associated with reduced microbial diversity and decreased abundance of some taxa, which may promote systemic immune dysregulation and mast cell activation [12]. Similarly, acne has been linked to gut microbial imbalances, potentially influencing systemic inflammation and sebum production through SCFA‐mediated pathways [9].

Another way of interaction is represented by the neuroendocrine system: gut microorganisms can stimulate neural pathways through the production of neurotransmitters. This mechanism may be particularly relevant in atopic dermatitis, where increased serotonin can trigger an itch‐scratch response [13].

Based on existing studies, this systematic review aims to synthesise the interconnections between gut microbiome and three prototypical skin conditions: Atopic Dermatitis (AD), Psoriasis (PSO) and Hidradenitis Suppurativa (HS). The primary objective is to identify the specific microbial signatures associated with each disease, which could potentially be targeted for therapeutic purposes. The secondary objective is to identify whether systemic therapy can influence gut microbiota composition and to explore any correlation between treatment‐induced microbial changes and clinical outcomes.

Several narrative and systematic reviews have recently considered the relationship between gut microbiota and individual inflammatory skin diseases, particularly AD and psoriasis. However, most available focus on single conditions and provide limited integration of functional and therapeutic data. The present systematic review aims to extend beyond prior literature by jointly analysing these three skin diseases with a unified methodological framework that integrates taxonomic, functional, and genetic causal‐inference evidence.

## 2 Materials and Methods

An electronic search was conducted using the MEDLINE database via PubMed to identify published articles on the gut microbiome and inflammatory skin diseases from inception to January 2025.

The search strings used were:

((Atopic Dermatitis OR Atopic Eczema OR Psoriasis OR Hidradenitis Suppurativa OR Acne Inversa) AND (Intestine OR Microbiota OR “Intestinal Microbiome” OR “Intestinal Microflora” OR “Gastrointestinal Microbiome” OR “Gut Microbiome”)). Moreover, the reference lists of the included studies were revised to identify further relevant studies.

**FIGURE 1:**
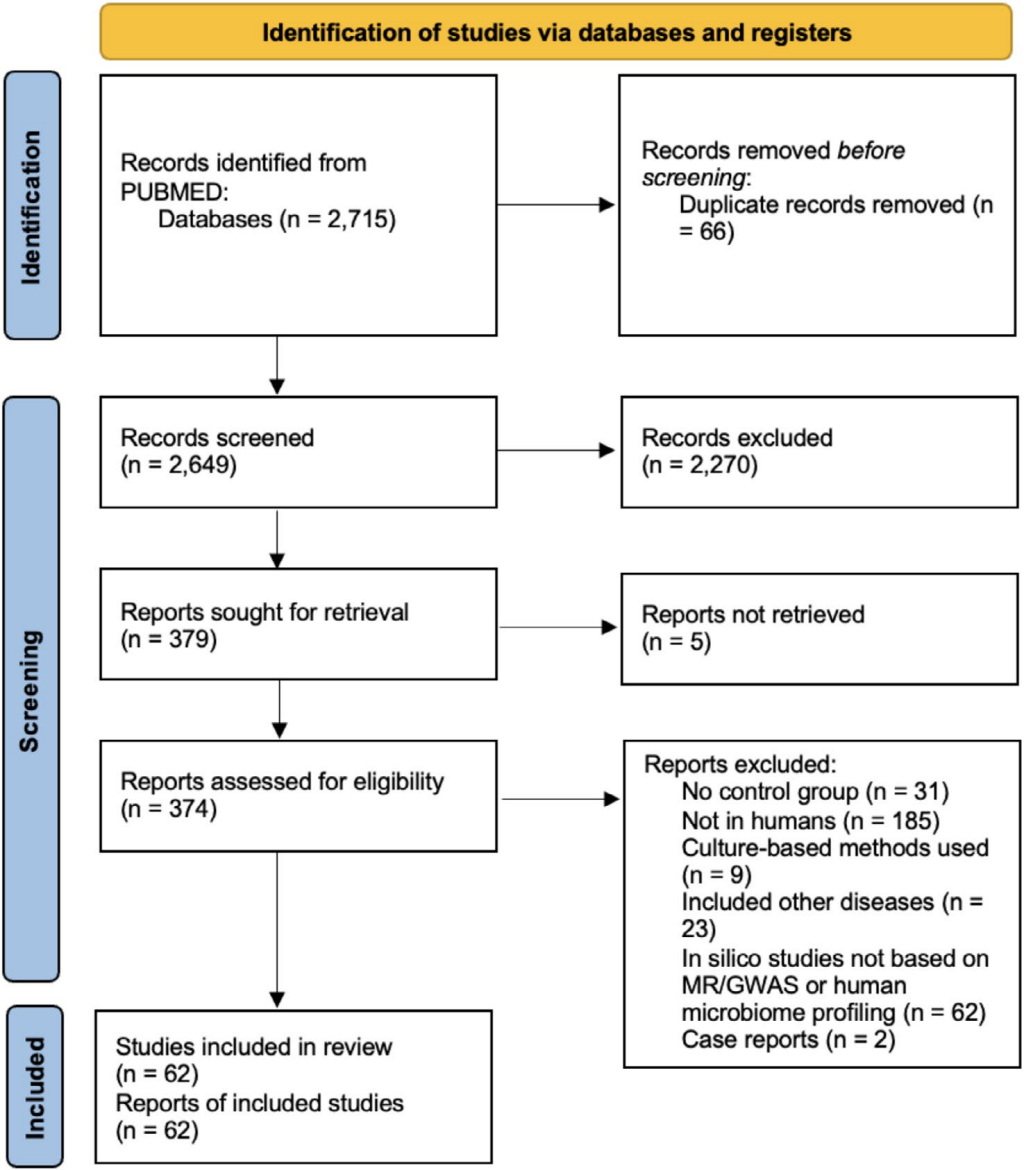
PRISMA 2020 flow diagram.

The work was conducted in accordance with the Preferred Reporting Items for Systematic Reviews and Meta‐Analyses (PRISMA 2020) statement (Figure 1).

Two reviewers (A.M. and G.N.) independently screened titles and abstracts for eligibility. Full texts of potentially relevant articles were subsequently assessed independently by the same reviewers. Discrepancies were resolved through discussion and, when necessary, consultation with a third author (A.V.M.). Before data extraction, A.M. designed a standardised data extraction form. The following information was extracted from the included articles: country, year, population, microbiome methodology (16S rRNA sequencing, shotgun metagenomics, RT‐PCR, Mendelian randomization), and main microbial findings.

Given the observational and heterogeneous nature of microbiome studies, no formal risk‐of‐bias assessment was performed, as standardised instruments are not structured to capture sources of variability in this field of research. However, during data synthesis we qualitatively considered microbiome‐specific sources of variability, including cohort size, age distribution, geographic background, sequencing methodology, and reporting of potential confounders such as antibiotic exposure or dietary factors.

The review protocol was not prospectively registered in a publicly accessible database (e.g., PROSPERO).

### 2.1 Eligibility Criteria

The inclusion criteria were intestinal microbiome studies investigating inflammatory skin diseases (atopic dermatitis, psoriasis and hidradenitis suppurativa), involving human subjects and written in English. Eligible studies included observational or interventional studies assessing gut microbiota composition using molecular techniques (next‐generation sequencing or real‐time PCR) on faecal samples or intestinal lavage, as well as human genetic studies exploring potential causal relationships between gut microbiota features and inflammatory skin diseases.

Studies were excluded if they studied conditions other than the three selected diseases (chronic spontaneous urticaria, food allergy, asthma, bullous pemphigoid, spondyloarthritis, among others), if the microbiota analysis was performed with cultural methods, or were conference abstracts, expert opinions, letters to the editor, reviews, or reported only one patient. For microbiome profiling studies, the absence of a healthy control group was an exclusion criterion.

## 3 Results

A total of 2715 records were identified from the PubMed database, of which 66 were initially removed as duplicates, leaving 2649 records for title and abstract screening. Of these, 2270 were excluded. The remaining 379 papers were sought for retrieval; 5 could not be retrieved. The remaining 374 reports were assessed for eligibility and 312 were excluded: 31 studies did not include controls, 185 did not involve humans, 9 used culture‐based methods for gut microbiota analysis, 23 included other diseases, 62 were in silico studies not based on MR/GWAS or human microbiome profiling, and 2 were case reports. A total of 62 papers were ultimately included in the systematic review.

The PRISMA flow diagram shows the systematic process we followed to include papers captured by our search (Figure 1).

Flowchart summarising the identification, screening, eligibility assessment, and inclusion of studies for the systematic review according to the PRISMA 2020 guidelines. A total of 2715 records were identified from PubMed, of which 62 studies met the inclusion criteria and were included in the final qualitative synthesis.

### 3.1 Atopic Dermatitis

Atopic dermatitis (AD) is a cutaneous inflammatory disease with a chronic‐relapsing course affecting approximately 20% of children and 2%–5% of adults worldwide [14]. Clinically, it is characterised by intense pruritus, eczema‐like eruptions with age‐specific distribution, varying degrees of xerosis and lichenification, especially in long‐standing cases.

It often represents the first manifestation of the “allergic triad”, which includes food allergy, asthma, and allergic rhinitis.

The pathophysiology is complex and multifactorial, involving a synergistic interaction between genetic predisposition, skin barrier dysfunction, immune dysregulation, and environmental factors. Two main models propose either immune dysfunction or barrier impairment as the initiating event.

This complex interaction between different pathogenic elements leads to an increase of Th2 and Th22 axes in acute lesions, while the Th2 to Th1 shift is fundamental in the acute‐chronic transition [15].

Therapeutic options include emollients, topical corticosteroids, calcineurin inhibitors and biologic agents such as Dupilumab (a monoclonal antibody targeting IL‐4 and IL‐13), as well as Tralokinumab and Lebrikizumab, which specifically target IL‐13. More recently, Nemolizumab, directed against IL‐31 receptor α, has shown efficacy in large phase 3 trials. Janus kinase (JAK) inhibitors such as Baricitinib, Upadacitinib and Abrocitinib represent efficient oral therapeutic options, acting by inhibiting intracellular signalling pathways involved in cytokine‐mediated inflammation.

Table 1 summarises the main findings in gut microbiota composition in AD patients.

**TABLE 1:** Summary of studies investigating the gut microbiota in atopic dermatitis (AD).

References	Nation, year	Findings in gut microbiota composition	Methodology	Cohort
Penders J [16]	Netherlands, 2006	Neither total bacterial profiles nor the type and proportion of *Bifidobacteria* in the faeces were associated with the development of AD. The development of IgE‐associated AD was however associated with colonisation by *E. coli*	RT‐PCR	26 ad 52 HC
Penders J [17]	Netherlands, 2007	Differences in gut microbiota composition precede the development of atopy. *E. coli* is only associated with AD and *Clostridioides. difficile* is associated with all atopic outcomes	RT‐PCR	957 infants, 30% of whom developed AD
Wang M [18]	Europe, 2008	Reduced alpha diversity in the early faecal microbiota of infants with AD during the first 18 months of life	16S rRNA gene–based fingerprinting	15 ad, 20 HC
Gore C [19]	UK, 2008	Presence of *Bifidobacterium pseudocatenulatum* in faeces is associated with AD and with exclusive formula‐feeding	16S rRNA gene–based fingerprinting	15 AD, 20 HC
Sjögren YM [20]	Sweden, 2009	Children who developed allergy (including AD) were significantly less colonised with *Lactobacilli group I*, *Bifidobacterium adolescentis* and *C. difficile* during the first 2 months	RT‐PCR	16 ad, 31 HC
van Nimwegen FA [21]	Netherlands, 2011	Colonisation by *C. difficile* at age 1 month is associated with AD through the first 6 to 7 years of life. It can probably be a mediator between the mode and place of delivery and the development of the disease	RT‐PCR	952 infants, 23.3% of whom developed AD at 2 years
Storrø O [22]	Norway, 2011	Patients with atopic sensitization have temporal variations of gut microbiota: lower levels of *Escherichia coli* at 4 months and 1 year, higher levels of *Bifidobacterium longum* at 1 year and lower levels of *Bacteroides fragilis* at 2 years. No association between colonisation pattern and AD	RT‐PCR	94 infants, 22 of whom with AD
Ismail IH [23]	Australia, 2012	A more diverse intestinal microbiota in the first week of life is associated with a reduced risk of subsequent AD in infants at high risk of allergic disease	16S rRNA gene–based fingerprinting	98 infants, 33 of whom developed AD at 1 year
Abrahamsson TR [24]	Sweden, 2012	Low intestinal microbiota diversity during the first month of life is associated with subsequent AD, particularly within the phylum Bacteroidetes and the genus *Bacteroides*	16S rRNA gene sequencing	20 ad, 20 HC
Nylund L [25]	Finland, 2013	Statistically significant differences in gut microbiota composition were found only at 18 months of age when comparing healthy and eczematous children. AD patients are found to have higher diversity, decreased relative abundances of Bacteroidetes and premature colonisation by *Clostridium* clusters IV and XIVa	16S rRNA gene microarray analysis	15 AD, 19 HC
West ce [26]	Australia, 2015	Reduced relative abundance of potentially immunomodulatory gut bacteria (*Ruminococcus*, *Enterobacteriaceae*) is associated with exaggerated inflammatory cytokine response to TLR‐ligands and subsequent development of AD. No evident associations with ‘protective’ genera as *Bifidobacterium* and *Lactobacillus*	16S rRNA gene sequencing	10 ad, 10 HC
Nylund L [27]	Finland, 2015	The severity of AD correlates inversely with microbiota diversity and with the abundance of butyrate‐producing bacteria (e.g., *Coprococcus eutactus* )	16S rRNA gene microarray analysis	28 ad, 11 HC
Ismail IH [28]	Australia, 2016	Early gut microbiota colonisation by *Bifidobacterium breve* is connected to a lower risk of developing AD, whereas *Bifidobacterium catenulatum* with a higher risk	RT‐PCR	41 ad, 76 HC
Lee E [29]	Korea, 2016	The relative abundance of Clostridia from infants with AD correlates with the age of disease onset and inversely correlates with blood eosinophil count	16S rRNA gene sequencing	12 ad, 12 HC
Song H [30]	Korea, 2016	Enrichment of a subspecies of the major gut species *Faecalibacterium prausnitzii* is strongly associated with AD, with reduced levels of butyrate and propionate	16S rRNA gene sequencing	90 ad, 42 HC
Zheng H [31]	China, 2016	Four genera are enriched in healthy infants: *Bifidobacterium, Megasphaera, Haemophilus* and *Streptococcus* spp.; and five genera are enriched in AD patients: *Escherichia/Shigella, Veillonella, Faecalibacterium, Lachnospiraceae incertae sedis* and *Clostridium XIVa*. *Bacteroides fragilis* and *Streptococcus salivarius* were less abundant in AD. *B. bifidum, B. longum* and *Bacteroides fragilis* were higher in healthy infants	16S rRNA gene sequencing	50 ad, 51 HC
Oh S [32]	Singapore, 2017	None of the major phyla and genera composing the gut microbiota showed a significant difference in relative abundance between control and AD communities. The sample size is small (6 AD and 6 controls). A higher occurrence of immunosuppressive motifs was observed in control communities, which were particularly enriched in the *Bifidobacterium longum* population	16S rRNA gene sequencing	6 AD, 6 HC
Mahdavinia M [33]	South Africa, 2017	Lack of statistically significant modification in alpha‐diversity and relative abundance of any taxa between the two groups. Small sample sizes (29 ad and 9 controls)	16S rRNA gene sequencing	29 AD, 9 HC
Lee MJ [34]	Korea, 2018	Bacterial cell amounts in the faeces were lower in infants with AD than in control infants. Although no specific taxa directly correlated with AD, whole‐metagenome analysis revealed differences in functional genes related to immune development	16S rRNA gene sequencing	63 ad, 66 HC
Li W [35]	China, 2019	No changes in alpha‐diversity of gut microbiota, whilst differences were recorded in skin and oral microbiota	16S rRNA gene sequencing	172 ad, 120 HC
Reddel S [36]	Italy, 2019	The gut microbiota of children with AD shows prevalence of species such as *Faecalibacterium, Oscillospira, Bacteroides, Parabacteroides and Sutterella* and reduction or complete absence of *Bifidobacterium, Blautia, Coprococcus, Eubacterium* and *Propionibacterium*	16S rRNA gene sequencing	19 ad, 18 HC
Melli LCFL [37]	Brazil, 2020	Children with AD harboured high levels of *C. difficile* , bifidobacteria and a low level of lactobacilli. *B. fragilis* and *Lactobacillus*, *E. coli* and *M. smithii* were lower in the healthy group	RT‐PCR	23 ad, 58 HC
Chan CWH [38]	China, 2020	The abundance of *Bifidobacterium* in the AD group was significantly higher than that in the control group	16S rRNA gene sequencing	24 ad, 24 HC
Galazzo G [39]	Germany, 2020	Longitudinal analysis of gut microbiota development in 440 children from 5 weeks through 6–11 years: lower microbial diversity associated with AD development and allergic sensitization. *Lachnobacterium* and *Faecalibacterium* were significantly decreased throughout infancy among children who developed AD	16S rRNA gene sequencing	440 infants, 25% of whom developed AD
Park YM [40]	Korea, 2020	Low levels of *Streptococcus* and high amounts of *Akkermansia* were evident in transient AD cases, and low *Clostridium, Akkermansia* and high *Streptococcus* were found in persistent AD. *Streptococcus* and *Clostridium* positively and negatively correlated with SCORAD score, respectively. Transient AD cases showed lower butyrate and valerate levels than healthy and persistent AD infants	16S rRNA gene sequencing	26 persistent AD, 22 transient AD and 84 HC
Ta LDH [41]	Singapore, 2020	Allergen‐sensitised AD infants have an aberrant developmental trajectory of the gut microbiome. The key signatures are an enrichment of *E. coli* and *Klebsiella pneumoniae* , a depletion of *Bacteroides fragilis* and low SCFA‐producing bacteria	16S rRNA gene sequencing	33 ad, 30 HC
Kingkaw A [42]	Thailand, 2020	No significant differences were found between the healthy and AD group. Eight significant proteins were uniquely expressed in the AD group, for example triosephosphate isomerase in *Bifidobacteriaceae* and demethylmenaquinone methyltransferase in *Bacteroides*	Metaproteomics	7 ad, 11 HC
Ye S [43]	China, 2021	Patients with AD have lower alpha‐diversity. *Bacteroidaceae* and *Porphyromonadaceae* can act as possible biomarkers associated with AD. *Clostridium* and *Prevotella* (SCFA‐producing bacteria) are lower in the AD group	16S rRNA gene sequencing	44 ad, 49 HC
Hu C [44]	Netherlands, 2021	The alpha‐diversity of gut microbiota is associated with a decreased risk of AD. The association of relative abundance and functional pathways is less consistent	16S rRNA gene sequencing	1140 infants, 7% of whom developed AD
Fan X [45]	China, 2022	Enrichment or reduction of certain gut microbiota in mother‐offspring pairs was associated with an increased risk of AD in infants and toddlers. No differences in diversity between AD and healthy patients at different ages (birth – 6 months—1 year—2 years)	16S rRNA gene sequencing	10 AD, 26 HC
Jiang Z [46]	Korea, 2022	A machine learning technique can classify AD with high precision (0.70) and recall (0.88) through the analysis of 35 genes and 50 microbial features. *Akkermansia* can affect the prediction the most	16S rRNA gene sequencing	88 ad, 73 HC
Lee M [47]	Korea, 2022	The diversity of gut microbiota in moderate to severe AD decreases compared to the non‐AD group at only 6 months. *Bifidobacterium* is not associated with AD, whereas persisting facultative anaerobes and limited *B. fragilis* colonisation with age are observed	16S rRNA gene sequencing	234 ad, 112 HC
Hoskinson C [48]	Canada, 2023	Decreases in *Anaerostipes hadrus, Fusicatenibacter saccharivorans, Eubacterium hallii and Blautia wexlerae* and increases in *Eggerthella lenta, E. coli, Enterococcus faecalis, Clostridium innocuum* and *Tyzzerella nexilis* are typical of allergic diseases including AD. Moreover, a core set of functional and metabolic imbalances was identified to be a significant mediator between microbiota maturation at age 1 year and allergic diagnoses at age 5 years	Shotgun metagenomic sequencing	282 ad, 523 HC
Jin Q [49]	China, 2023	Seven causal associations from bacterial taxa to AD were identified by the IVW method (part of a two‐sample Mendelian randomization analysis). The genera *Dialister* and *Prevotella* were associated with a higher risk of AD	Genome‐wide association study (GWAS)‐based Mendelian randomization (MR) analysis	10 277 ad, 278795 HC
Liu X [50]	China, 2023	39 genera showed significant differences in the gut microbiota between AD patients and controls: an enrichment of *Lactobacillus* and *Streptococcus* and a reduction of *Eubacterium* in AD is observed. Moreover, elevated levels of IgE in AD are related to specific microbes	Shotgun metagenomic sequencing	68 ad, 77 HC
Mao R [51]	China, 2023	The common probiotics such as family *Bifidobacteriaceae* and genus *Bifidobacterium* have a protective effect on AD. The reverse MR analysis shows a bidirectional causal effect between AD and genus *Eubacterium brachy* group	GWAS‐based MR analysis	7021 ad, 198740 HC
Yang L [52]	China, 2024	Increased *Firmicutes/Bacteroidetes* ratio, decreased *Bifidobacterium* and expanded *Faecalibacterium* are identified among AD cases. After 16 weeks of Dupilumab treatment, improvement in beta‐diversity, increased colonisation of *Bifidobacterium*, *Ruminococcus gnavus* and *Coprococcus* was observed, independently of clinical indicators	16S rRNA gene sequencing	44 AD, 27 ad patients who received Dupilumab, 48 HC
Zhong Y [53]	China, 2024	Bidirectional two‐sample MR showed that *FamilyXIII* is associated with increased risk for AD. The genus *Dialister* has a protective role. The reverse two‐sample MR analysis indicated no reverse causality between inflammatory skin diseases and the identified gut microbiota	GWAS‐based MR analysis	13 473 ad, 336589 HC

*Note:* This table summarises 38 studies evaluating gut microbiota composition in patients with atopic dermatitis (AD), two of which also included psoriasis cohorts. For each study, the country, year of publication, main findings, analytical methodology, and cohort characteristics are reported.

Abbreviations: 16S rRNA gene sequencing, amplicon‐based sequencing of the 16S ribosomal RNA gene; AD, atopic dermatitis; GWAS, genome‐wide association study; HC, healthy controls; MR, Mendelian randomization; RT‐PCR, real‐time polymerase chain reaction; SCFA, short‐chain fatty acids.

Since the early 2000s, greater evidence has highlighted the role of gut microbiota in the pathogenesis of AD.

Gut microbiota plays a vital role in regulating innate and adaptive immunity, influencing the development of allergic diseases. Reduced microbial diversity and richness in early childhood are associated with abnormal immune maturation, insufficient Th1 cell induction and failure to suppress Th2 responses—hallmarks of allergic diseases.

Reduced alpha diversity in gut microbiota has been reported in association with increased AD risk and greater disease severity [18, 19, 27, 36, 44]. These findings support the hygiene hypothesis revised, as proposed by Wold [54]. The hygiene hypothesis suggests that exposure to a wide range of infectious agents during the first years of life can protect against allergic diseases.

However, some studies report no significant diversity differences between AD and healthy controls at certain ages. By adulthood, gut microbiota tends to stabilise and variations in diversity may reflect long‐term compensatory changes rather than the dramatic shifts seen in infancy [43]. Data on adults remain sparse and are flagged as an area needing further investigation.

Specific microbial taxa have been associated with AD. Infants with AD often show decreased levels of *Lactobacillus* spp., *Bifidobacterium* spp., *Akkermansia muciniphila* , and butyrate‐producing bacteria like *Faecalibacterium prausnitzii* and *Coprococcus eutactus* .

Multiple studies consistently report a reduced abundance of *Lactobacillus* species in paediatric AD patients compared to healthy controls. This can be linked to a weakened gut epithelial barrier, promoting systemic inflammation through translocation of antigens and toxins from the intestinal lumen [22].

*Bifidobacterium* spp. contribute to vitamin production, improvement of food ingredients digestion, and immune system stimulation. Lower levels of bifidobacteria in AD patients are reported, with levels of *B. breve* and *B. longum* positively correlating with symptom severity [28, 31, 37, 38, 51]. *B. longum* is a promising probiotic strain for alleviating AD symptoms. It has been shown to upregulate tryptophan metabolism and increase the production of indole‐3‐carbaldehyde (I3C), which activates the aryl hydrocarbon receptor (AhR) pathway to suppress aberrant Th2 immune responses [55].

*F. prausnitzii* emerged as one of the most influential taxa in predictive models for microbiome differences between AD and non‐AD individuals. Its abundance correlates with disease severity and gut microbiota maturity in early childhood [30].

*Akkermansia muciniphila* contributes to gut health by degrading mucin and producing metabolites like SCFAs. In AD, altered *A. muciniphila* abundance could affect the availability of nutrients for other bacterial species, as well as alter SCFA production and oxidative phosphorylation.

Conversely, high levels of *Escherichia coli* , *Clostridioides difficile*, and certain *Bifidobacterium* species have been associated with AD.

*E. coli* , an early coloniser of the infant gut, shows strain‐ and population‐specific associations with AD. An increased abundance of this bacterial species in infants at 1 month of age who later developed AD was reported in a large Birth Cohort Study [17]. This finding is consistent with the hypothesis that LPS originating from *E. coli* might play a role in regulating the immune system through intestinal epithelial cells.

West et al. in 2015 highlighted how *E. coli* strains like *Escherichia coli* Nissle 1917 can have immunomodulatory properties, emphasising the need to distinguish between pathogenic and commensal strains [26].

*Clostridium* species are often present in higher abundance in individuals with AD. *C. difficile* is found at slightly higher prevalence in children with AD in some studies and may be linked to persistent inflammation through toxin‐mediated epithelial disruption [17, 21, 37].

Therapeutic interventions also appear to influence the gut microbiota. There is only one study investigating the effect of biologic drugs on gut microbiota in AD. Dupilumab therapy improved pre‐treatment gut microbiota dysbiosis, including increasing the abundance of beneficial taxa like *Bifidobacterium* and SCFA‐producing bacteria, independently of its effects on skin inflammation [52].

Variability in findings, particularly concerning specific taxa such as *Faecalibacterium* and *Bifidobacterium*, highlights the influence of study design, sample size, and population diversity. Moreover, because AD primarily affects infants undergoing rapid gut microbiota development, combining data from paediatric and adult cohorts may obscure age‐specific patterns.

### 3.2 Psoriasis

Psoriasis is a chronic inflammatory skin disease affecting 2%–3% of the global population, with 30% experiencing severe forms, characterised by epidermal proliferation and infiltration of inflammatory immune cells. It typically presents as raised, well‐demarcated, erythematous, scaly plaques due to epidermal hyperproliferation and parakeratosis [56].

The TNFα‐IL‐23‐Th17 inflammatory pathway plays a central role in pathogenesis, as supported by the success of target biologics that achieve high clinical response rates [57].

Moreover, the metabolic aspects of chronic Th‐1 and Th‐17 inflammation in psoriasis have been associated with systemic comorbidities such as obesity, diabetes, and atherosclerosis.

Diagnosis of psoriasis is clinical and severity guides treatment: mild to moderate disease is managed with topical therapy such as corticosteroids and vitamin D3 analogues.

Phototherapy and systemic agents are used in more severe cases.

Currently available biologic treatments for moderate‐to‐severe psoriasis target specific components of the immune system with high efficacy and favourable safety profiles. The earliest class is TNF‐α inhibitors, such as Adalimumab, which remain the mainstay in many treatment algorithms. Next came IL‐12/23 inhibitors, with Ustekinumab blocking the p40 subunit shared by IL‐12 and IL‐23. More recently, IL‐23 p19 inhibitors such as Guselkumab, Risankizumab and Tildrakizumab have offered selective immunomodulation with excellent clinical profiles. The IL‐17 is also a potential target: biologics, including Secukinumab, Ixekizumab, Brodalumab, and Bimekizumab, target IL‐17A or both IL‐17A and IL‐17F.

Table 2 summarises the main findings in gut microbiota composition in psoriasis patients.

**TABLE 2:** Summary of studies investigating gut microbiota alterations in psoriasis (PSO).

References	Nation, year	Findings in gut microbiota composition	Methodology	Cohort
Scher JU [58]	Spain, 2015	Lower alpha‐diversity in psoriasis and psoriatic arthritis patients compared to healthy controls. Reduced relative abundance of *Parabacteroides* and *Coprobacillus*	16S rRNA gene sequencing	15 PSO, 17 HC
Doaa M [59]	Egypt, 2016	Psoriasis patients have a higher F/B ratio, which correlates with PASI score. Actinobacteria, particularly *Bifidobacterium*, are reduced	RT‐PCR	45 PSO, 45 HC
Eppinga H [60]	Netherlands, 2016	*F. prausnitzii* is significantly decreased and *E. coli* is significantly increased in the gut of psoriasis patients	RT‐PCR	29 PSO, 33 HC
Tan L [61]	China, 2018	The abundance of *A. muciniphila* was significantly reduced in patients with psoriasis	16S rRNA gene sequencing	14 PSO, 14 HC
Chen YJ [62]	Taiwan, 2018	Psoriasis patients have an increased abundance of Firmicutes and decreased Bacteroidetes. *Ruminococcus* and *Megasphaera* are the top two genera of discriminant abundance in psoriasis	16S rRNA gene sequencing	32 PSO, 64 HC
Huang L [63]	China, 2019	Psoriasis is associated with increased abundance of Bacteroidetes and decreased Firmicutes (inverted F/B ratio). At a genus level, *Bacteroides, Sutterella* and *Parabacteroides* are more abundant in psoriasis, while *Bifidobacterium* is reduced. *Veillonella* correlates with inflammatory markers like C‐reactive protein	16S rRNA gene sequencing	35 PSO, 27 HC
Shapiro J [64]	Israel, 2019	Patients with psoriasis have an increase in Firmicutes and Actinobacteria. At a species level, *Ruminococcus gnavus, Dorea formicigenerans* and *Collinsella aerofaciens* are increased; *Prevotella copri* and *Parabacteroides distasonis* are decreased	16S rRNA gene sequencing	24 PSO, 22 HC
Hidalgo‐Cantabrana C [65]	Spain, 2019	Increase in Actinobacteria and Firmicutes in the psoriasis group, whereas Bacteroidetes and Proteobacteria are reduced. Increase in the Firmicutes/Bacteroidetes ratio. *Ruminococcus*, *Blautia*, *Collinsella*, *Dorea* and *Bifidobacterium* are reduced	16S rRNA gene sequencing	19 PSO, 20 HC
Yeh NL [66]	Taiwan, 2019	Secukinumab treatment causes more profound alterations in the gut microbiome, including an increase in Proteobacteria and decreases in Bacteroidetes and Firmicutes, than Ustekinumab treatment. Moreover, Secukinumab increases the relative abundance of *Pseudomonadaceae* and *Enterobacteriaceae*	16S rRNA gene sequencing	34 PSO (24 Secukinumab, 10 Ustekinumab), 12 HC
Dei‐Cas I [67]	Argentina, 2020	Increase in Firmicutes and reduction of Bacteroidetes in psoriasis. *Faecalibacterium* and *Blautia* genus were higher in psoriasis, while *Bacteroides* and *Paraprevotella* in controls. Moderate‐to‐severe psoriasis patients have lower biodiversity than mild	16S rRNA gene sequencing	55 PSO, 27 HC
Yegorov S [68]	Kazakhstan, 2020	Psoriasis was associated with alterations in gut Firmicutes, including elevated *Faecalibacterium* and decreased *Oscillibacter* and *Roseburia* abundance, as well as significantly elevated gut IL‐1α	16S rRNA gene sequencing	20 PSO, 20 HC
Wang X [69]	China, 2021	*Negativicutes*, *Bacilli*, *Selenomonadales*, *Lactobacillus*, *Veillonellaceae* and *Megamonas* are more represented in psoriasis patients. *Romboutsia* is the key type in the healthy subjects. At a genus level, a lower relative abundance of *Romboutsia* and a higher abundance of *Megamonas* are recorded	16S rRNA gene sequencing	20 PSO, 20 HC
Xiao S [70]	China, 2021	No difference in alpha‐diversity. Increased abundance of phyla Firmicutes, Actinobacteria and Verrucomicrobia and genera *Faecalibacterium, Bacteroides, Bifidobacterium, Megamonas* and *Roseburia* and a decreased abundance of the phyla Bacteroidetes, Euryarchaeota and Proteobacteria and genera *Prevotella*, *Alistipes* and *Eubacterium*	16S rRNA gene sequencing	30 PSO, 15 HC
Zhang X [71]	China, 2021	*Faecalibacterium* and *Megamonas* are increased in patients with psoriasis. Microbiome variations are associated with levels of many cytokines as interleukin‐2 receptor	16S rRNA gene sequencing	30 PSO, 30 HC
Todberg T [72]	Denmark, 2022	Psoriasis patients have reduced richness and diversity in the community composition of metagenomic species. Higher abundances of *Ruminococcus torques* and reduced *Faecalibacterium* spp. Functional analysis revealed altered metabolic pathways, such as increased glutamate degradation	Shotgun metagenomic sequencing analysis	53 PSO, 73 HC
Rungjang A [73]	Thailand, 2022	No statistically different proportions of dominant phyla between psoriasis patients and controls. The abundance of *Lactobacillus* and *Ruminococcus* was significantly increased in patients with psoriasis after NBUVB treatment, particularly in the responders	Shotgun metagenomic sequencing analysis	13 PSO, 9 HC
Mao R [51]	China, 2023	There is a unidirectional causal relationship between gut microbiota and psoriasis, as concluded by a bidirectional MR analysis. Only 5 genera are associated with psoriasis: genus *Odoribacter* has a protective effect, whilst others as *Lactococcus* and *Eubacterium fissicatena* group are risk factors	GWAS‐based MR analysis	4510 PSO, 212242 HC
Wu R [74]	China, 2024	There is a causal relationship between psoriasis and the family *Veillonellaceae* and the genera *Candidatus soleaferrea* and *Eubacterium fissicatena* group	GWAS‐based MR analysis	9267 PSO, 364071 HC
Xiao Y [75]	China, 2024	Reduced *Eubacterium rectale* in both Psoriasis and Psoriatic arthritis patients, with the latter exhibiting even lower levels of *E. rectale* than the former. Additionally, two *Alistipes* species were also depleted in psoriatic patients	Shotgun metagenomic sequencing	44 PSO, 25 HC
Zhao H [76]	China, 2024	Decreased Bacteroidetes and increased Firmicutes and Actinobacteria are seen in psoriasis patients. IL‐17A inhibitor treatment alters the gut microbiota and tends to shift it toward a healthy state	16S rRNA gene sequencing	14 PSO, 10 HC
Zhong Y [53]	China, 2024	*Eubacterium fissicatena group* is associated with an increased risk for psoriasis. The reverse S2MR analysis indicated no reverse causality between inflammatory skin diseases and the identified gut microbiota	GWAS‐based MR analysis	9267 PSO, 364071 HC
Cozma EC [77]	Romania, 2024	Lower levels of Firmicutes and *Enterobacteriaceae* in the psoriasis group	RT‐PCR	10 PSO, 10 HC

*Note:* This table summarises 22 studies evaluating gut microbiota composition in patients with psoriasis, including two that also examined atopic dermatitis (AD) and one overlapping with hidradenitis suppurativa (HS). For each study, the country, year, main microbial findings, analytical methodology, and cohort characteristics are reported.

Abbreviations: 16S rRNA gene sequencing, amplicon‐based sequencing of the 16S ribosomal RNA gene; AD, atopic dermatitis; F/B ratio, Firmicutes/Bacteroidetes ratio; GWAS, genome‐wide association study; HC, healthy controls; HS, hidradenitis suppurativa; MR, Mendelian randomization; PASI, Psoriasis Area and Severity Index; RT‐PCR, real‐time polymerase chain reaction.

Interest in the role of gut microbiota in psoriasis pathogenesis has emerged more recently than in AD.

Reduced alpha‐diversity has been reported across multiple studies included in this systematic review [58, 72]. Yeh et al. [66] reported no significant changes in alpha‐diversity following treatment with Secukinumab or Ustekinumab. However, significant alterations in beta diversity were observed in patients receiving Secukinumab, suggesting treatment‐related shifts in microbial communities.

At the phylum level, psoriasis is consistently associated with a reduction in Bacteroidetes. This shift alters the Firmicutes/Bacteroidetes ratio, which is critical for maintaining normal intestinal homeostasis. *Bacteroides* are implicated in the regulation of T‐helper 17 (Th17) cells, and their depletion may contribute to Th17 axis dysregulation and hyperactivation of psoriasis‐related immune pathways [67, 76].

The phylum Actinobacteria, generally associated with anti‐inflammatory functions and mucosal homeostasis, shows variable trends across studies: some report decreased abundance in psoriasis patients [59, 76], while others report increases or no significant changes [64, 65, 75].

Several SCFA‐producing taxa are consistently reduced. *F. prausnitzii* , a major butyrate producer with well‐known anti‐inflammatory properties, is depleted in psoriasis patients [60, 67, 77]. Similar reductions have been reported in IBDs, a common comorbidity of psoriasis. *F. prausnitzii* depletion has been proposed as a biomarker of intestinal dysbiosis and a potential target for next‐generation probiotic development.

*Akkermansia muciniphila* , previously discussed, is also significantly reduced in psoriasis [61, 63, 75]. Its depletion may compromise mucus layer integrity and favour low‐grade inflammation.

*Prevotella copri* is reported to be reduced in one study [64]. This genus is associated with carbohydrate metabolism, such as fibre fermentation, and anti‐inflammatory pathways.

*Alistipes* species, involved in gut health and SCFA production, are also underrepresented in psoriasis, further contributing to a pro‐inflammatory gut environment.

A consistent reduction of *Eubacterium rectale* has been reported [64, 75]. This is another key butyrate producer that colonises the mucus layer and sustains epithelial energy metabolism. Its reduction impairs the production of SCFAs, weakening gut barrier function and potentially exacerbating systemic inflammation [78].

Finally, *Parabacteroides distasonis*, associated with protective gut functions, is significantly reduced in psoriasis patients [64].

### 3.3 Hidradenitis Suppurativa

Hidradenitis suppurativa (HS) is an inflammatory disease characterised by chronic deep‐seated nodules, abscesses, fistulae, sinus tracts and scars in intertriginous areas. Its global prevalence ranges from 0.0003% to 4% [79]. HS significantly impairs quality of life due to pain and excessive scarring, eventually leading to disfigurement.

The disease primarily affects the pilosebaceous‐apocrine unit, beginning with follicular occlusion and rupture, followed by chronic inflammation and sinus tract formation [80].

TNFα and IL‐17 are key inflammatory mediators, which link to metabolic comorbidities of HS like obesity, dyslipidemia and metabolic syndrome.

Treatment requires a multimodal approach, including antibiotics, corticosteroids, immunosuppressants, and biologics, as well as surgery for difficult cases.

Adalimumab was the first biotechnology drug approved for HS, though with variable efficacy in daily practice. More recently, Secukinumab (anti‐IL‐17A) and Bimekizumab (targeting IL‐17A and IL‐17F) have been approved for HS, due to the promising results in phase 3 trials.

Table 3 summarises the main findings in gut microbiota composition in HS patients.

**TABLE 3:** Summary of studies investigating gut microbiota alterations in hidradenitis suppurativa (HS).

References	Nation, year	Findings in gut microbiota composition	Methodology	Cohort
Eppinga H [60]	Netherlands, 2016	HS shows a distinct gut microbiota profile compared to psoriasis and IBD as there is neither a significant depletion of *F. prausnitzii n*or an increase in *E. coli*	RT‐PCR	17 HS, 33 HC
Lam SY [81]	Netherlands, 2021	No difference in alpha or beta diversity. *Robinsoniella peoriensis* and *Sellimonas* are more common in HS patients	16S rRNA gene sequencing	17 HS, 20 HC
Kam S [82]	USA, 2021	Lower relative abundance of Firmicutes in HS. *Bilophila* and *Holdemania* are among the more abundant genera in HS, whereas *Lachnobacterium* and *Veillonella* are reduced	16S rRNA gene sequencing	3 HS, 3 HC
Tatian A [83]	Australia, 2022	After 12 weeks of Adalimumab therapy, HS patients show higher abundances of *Bifidobacterium*, *Bacteroides*, *Agathobacter*, *Prevotella_9*, *Faecalibacterium* and *Blautia*, as well as increased SCFAs	16S rRNA gene sequencing	10 HS, 6 HC
McCarthy S [84]	Ireland, 2022	Alpha‐diversity is significantly lower. Elevated levels of *Ruminococcus gnavus* and *Clostridium ramosum* are among the greatest differences. Pathways involved in galactarate and glucarate degradation are more enriched in the gut microbiota of HS patients (linked to systemic inflammation)	16S rRNA gene sequencing	59 HS, 30 HC

*Note:* This table summarises five studies evaluating gut microbiota composition in patients with hidradenitis suppurativa (HS), including one study overlapping with psoriasis (PSO). For each study, the country, year, main microbial findings, analytical methodology, and cohort characteristics are reported.

Abbreviations: RT‐PCR, real‐time polymerase chain reaction; 16S rRNA gene sequencing, amplicon‐based sequencing of the 16S ribosomal RNA gene; SCFA, short‐chain fatty acids; HS, hidradenitis suppurativa; PSO, psoriasis; HC, healthy controls.

Studies on gut microbiota are expanding in number, but data on the influence of microbiota in HS remain limited.

McCarthy et al. [84] reported reduced alpha‐diversity in faecal samples from 59 patients with HS, whereas Lam SY et al. [81] found no difference, although their study considered a smaller cohort.

*Ruminococcus gnavus* is significantly more abundant in HS patients than in healthy controls. This aligns with data obtained with IBD, such as Crohn's disease, and may reflect shared inflammatory pathways.

To date, only one study identified a significant enrichment of *Streptococcus* spp. in the gut microbiota of HS patients, suggesting a possible role in disease‐associated dysbiosis [84]. While some *Streptococcus* species are normal gut residents, their overrepresentation—especially when combined with reduced levels of beneficial taxa like *Faecalibacterium prausnitzii* or *Bifidobacterium* spp.*—*is a recurrent feature of dysbiosis. Elevated *Streptococcus* has been associated with inflammatory and metabolic conditions including Crohn's disease [85, 86], irritable bowel syndrome (IBS) [87] and colorectal cancer [88]. Its expansion may reflect a shift toward lactate‐dominated fermentation, altered immune stimulation (via TLRs), and impaired cross‐feeding to butyrate producers, thereby amplifying inflammation and disrupting gut homeostasis.

Following treatment with Adalimumab, a higher relative abundance of *Prevotella* spp. and *Faecalibacterium prausnitzii* was observed in HS patients. Both taxa positively correlated with faecal propionate levels. In particular, the pathways driving the changes in SCFAs concentrations were bifidobacterium‐shunt, amino acid biosynthesis pathways (urea cycle and L‐citrulline biosynthesis), formaldehyde assimilation and oxidation pathways [83].

### 3.4 Stratified Synthesis

Given the marked heterogeneity of included studies, findings were synthesised narratively and interpreted in a stratified manner according to age group, analytical methodology, disease severity and treatment status.

A qualitative, stratified synthesis of the direction and consistency of reported gut microbiota alterations across the three diseases is summarised in Table 4.

**TABLE 4:** Direction and consistency of gut microbiota alterations across inflammatory skin diseases.

Feature/Taxon	AD	PSO	HS	Confidence
Alpha‐diversity	↓	↓	↓/↔	Moderate
*Faecalibacterium prausnitzii*	↓	↓	↔/↓	Moderate (AD, PSO), Low (HS)
Bifidobacterium spp.	↓	↔/↓	↔	Moderate
*Akkermansia muciniphila*	↓	↓	↔	Moderate
SCFA‐producing taxa (overall)	↓	↓	↓/↔	Moderate
*Ruminococcus gnavus*	↔/↑	↑	↑	Moderate
*Escherichia coli*	↑	↑	↔	Low–Moderate
*Eubacterium rectale*	↓	↓	n.a.	Moderate
Firmicutes/Bacteroidetes ratio	↑/↔	↑	n.a.	Low–Moderate
Functional pathways (SCFA, AA metabolism)	Altered	Altered	Altered	Low–Moderate

*Note:* ↑, increased relative abundance; ↓, decreased relative abundance; ↔, inconsistent change. Arrows indicate the predominant direction of reported associations across heterogeneous studies and do not imply causality.

Abbreviation: n.a., not adequately studied.

## 4 Conclusions and Perspectives

Recent advances in molecular biology have expanded our understanding of the gut‐skin axis, with numerous studies linking intestinal microbiota alterations to inflammatory skin diseases such as AD, psoriasis, and HS. However, several knowledge gaps persist, particularly regarding the adult population in AD research. Most available studies are paediatric‐focused, leaving the gut microbiota dynamics in adult AD largely unexplored and highlighting the need for more age‐specific investigations.

In psoriasis, research has shifted from a simple taxonomic profile toward a more functional perspective, incorporating metagenomic and metabolomic insights. Studies now explore microbial metabolic pathways, such as SCFA production, tryptophan metabolism, and amino acid biosynthesis, to unravel mechanisms through which gut microbes influence systemic inflammation and immune regulation. This functional understanding represents a key step toward identifying microbiota‐derived biomarkers and therapeutic targets. Across conditions, a similar dysbiotic pattern emerges, characterised by decreased abundance of *Faecalibacterium prausnitzii* and other SCFA‐producing bacteria, along with reduced microbial diversity. This shared microbial signature may be associated with common mechanisms of systemic inflammation and immune dysregulation along the gut–skin axis.

Evidence linking systemic biologic therapies to changes in gut microbiota remains limited across all three diseases. These observations should be considered exploratory and of low certainty, as they derive from small cohorts and may be confounded by prior or concomitant medications, dietary factors, and regression to the mean.

This review has limitations. No formal risk‐of‐bias assessment was performed, which limits the ability to weigh the strength of individual studies. Moreover, heterogeneity in study design, populations, age groups, and analytical approaches complicates direct comparison across studies. In addition, the review protocol was not prospectively registered.

Studies in HS remain limited but reduced microbial diversity and enrichment of *Ruminococcus gnavus* and *Streptococcus* spp. are frequently reported, resembling patterns seen in IBD.

Beyond summarising existing evidence, this systematic review offers an integrated overview of gut microbiota alterations across three epidemiologically frequent skin diseases. This cross‐disease perspective, accounting for functional and causal‐inference data, highlights both shared dysbiotic signatures—particularly the depletion of SCFA‐producing taxa—and disease‐specific microbial alterations.

Data on the impact of systemic therapy on gut microbiota composition are currently limited and derive from only a few studies for each disease; further research is needed to clarify this relationship.

Establishing a causal relationship between gut microbiota alterations and disease remains challenging. Generally, the primary method for determining causality is a randomised controlled trial (RCT). RCTs are complex to complete, and it can be difficult to quantify the impact of single factors. Mendelian randomization (MR) offers a complementary approach: by leveraging genetic variants as proxies for exposures, MR can help infer potential causal effects, avoiding the influence of reverse causality and minimising confounding factors [53].

Recent MR studies suggest unidirectional causality between specific bacterial taxa and psoriasis or AD, supporting the hypothesis that gut microbiota alterations may precede and contribute to disease onset [49, 51]. These findings warrant further validation but point to MR as a valuable tool for strengthening causal inference in microbiota research.

Artificial intelligence (AI) and machine learning are also transforming the field. By integrating complex datasets such as metagenomics, metabolomics, and transcriptomics, AI models can improve disease classification, biomarker discovery, and personalised therapeutic targeting. For instance, Jiang et al. successfully combined transcriptomic and microbial data to diagnose AD with high precision and recall using supervised machine learning approaches [46].

This systematic review aims to make general and uniform considerations and tries to address existing biases by selecting studies that used highly replicable technologies (such as 16S rRNA sequencing and metagenomic shotgun sequencing) and included different ethnic groups. Large and well‐designed cohorts like the CHILD study are crucial to add evidence to this field of research [48].

## 5 Major Open Questions

Despite accumulating evidence linking gut dysbiosis to inflammatory skin diseases, several key questions remain.

The first concerns causality and mechanisms: it is still unclear how specific microbial taxa and metabolites promote systemic inflammation and cutaneous immune dysregulation. This gap can be addressed through integrated multi‐omic studies that combine metagenomic, metabolomic, and host transcriptomic data to uncover functionally relevant microbial pathways.

Another critical question involves age‐related patterns, particularly in adult AD, where evidence remains scarce compared with paediatric cohorts. Longitudinal cohorts of large sizes are needed to clarify whether microbial signatures evolve or persist with age and disease duration.

Therapeutic modulation of the microbiome is also largely unexplored. Robust randomised controlled trials are necessary to determine whether dietary interventions, probiotics, or microbiota‐based therapies can significantly impact disease course or treatment outcomes.

Finally, the application of AI and machine learning presents promising avenues for integrating complex datasets, enhancing disease classification, and identifying predictive biomarkers. Developing interpretable, standardised, and multi‐ethnic models will be essential to ensure reproducibility and clinical translation.

Collectively, addressing these open questions with the growing power of AI to integrate and interpret complex biological data will accelerate the development of microbiome‐supported diagnostics and therapeutics in dermatology.

## Author Contributions

Conceptualization: A.M., G.N.; Data acquisition and curation: A.M., M.D.M.; Methodology: A.M., G.N., E.B.; Writing and editing: A.M., M.D.M., G.N.; Supervision: E.B., A.V.M.; Writing – review and editing: A.V.M., G.N. All authors have read and approved the final manuscript.

## Funding

The Department of Pathophysiology and Transplantation, University of Milan, is funded by the Italian Ministry of Education and Research (MUR): Dipartimenti di Eccellenza Program 2023 to 2027.

## Ethics Statement

The authors have nothing to report.

## Conflicts of Interest

The authors declare no conflicts of interest.

## Data Availability

Data sharing not applicable to this article as no datasets were generated or analysed during the current study.
